# 52. PrEP Adherence and Discontinuation at a Pharmacy-Supported PrEP Program in Atlanta, GA

**DOI:** 10.1093/ofid/ofab466.052

**Published:** 2021-12-04

**Authors:** Hiba Yacout, Bradley L Smith, Shelbie Foster, Meredith Lora, Laris Niles-Carnes, Ziduo Zheng, Suprateek Kundu, Judah K Gruen, Valeria D Cantos

**Affiliations:** 1 Grady Health System, Macon, Georgia; 2 Emory University, Atlanta, Georgia

## Abstract

**Background:**

Pre-exposure prophylaxis (PrEP) is a highly effective biomedical strategy to decrease Human Immunodeficiency Virus (HIV) acquisition. Effectiveness of oral PrEP is linked to medication adherence. In 2018, Grady Health System (GHS) launched a PrEP program to increase PrEP access among un- and underinsured individuals living in metro Atlanta, Georgia. The purpose of this study is to determine PrEP medication adherence, PrEP discontinuation rates, and associated individual factors of patients enrolled during the first 18 months of the program’s implementation.

**Methods:**

A single-center, retrospective chart review was conducted on patients enrolled in the GHS PrEP program between June 1, 2018 and February 29, 2020 who received more than one monthly PrEP prescription. Adherence was estimated using the medication possession ratio (MPR). The primary outcome was mean adherence to PrEP. Secondary outcomes include rate of high percent adherence (MPR > 80%), median time of engagement in care, PrEP discontinuation rates, rates of PrEP re-engagement, and individual factors associated with PrEP discontinuation and low adherence.

**Results:**

This study included 154 patients, 70.8% of them were Black, 62.3% were cisgender men, 59.1% were uninsured, and the mean age was 34. The majority of patients identified as men who have sex with men (51.9%). Mean PrEP adherence was 89.2%; 77.3% of patients demonstrated a high rate of adherence. No individual or social factors were associated with low adherence, but younger age was associated with higher rates of PrEP discontinuation (p< 0.0061). At the end of the follow up period on October 30, 2020, 53.8% of patients were active in the program and 12.7% of those who discontinued had re-engaged with the program. The average length of program engagement was 9.8 months.

Table 1. Baseline socio-demographic characteristics (N=154)

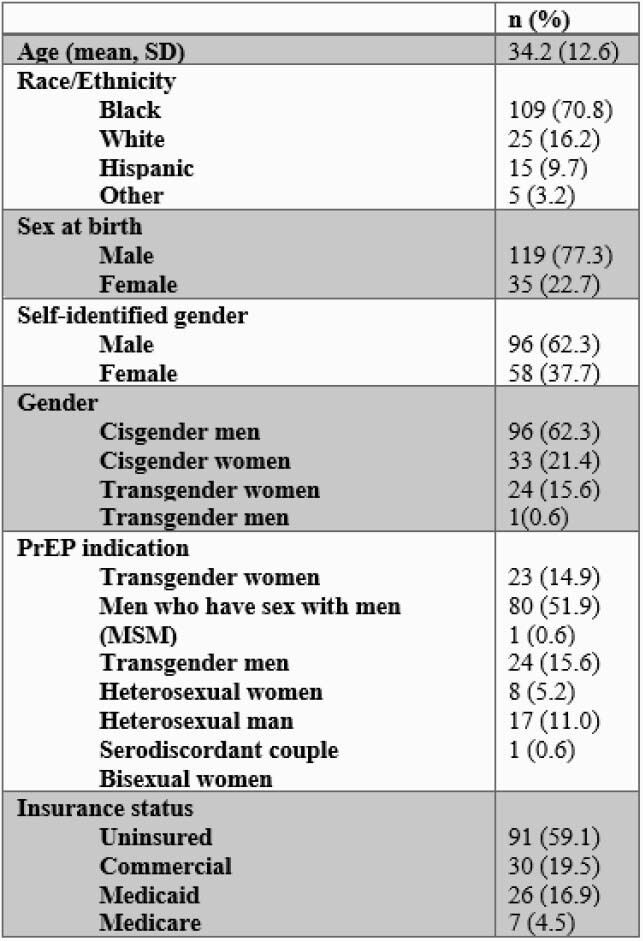

Table 2. PrEP Adherence and Discontinuation at the GHS PrEP Program from 2018 to 2020 (N=154)

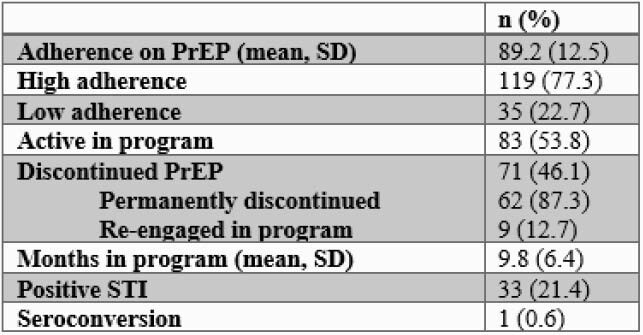

Table 4. Multivariate analysis of individual factors associated with PrEP discontinuation and low adherence

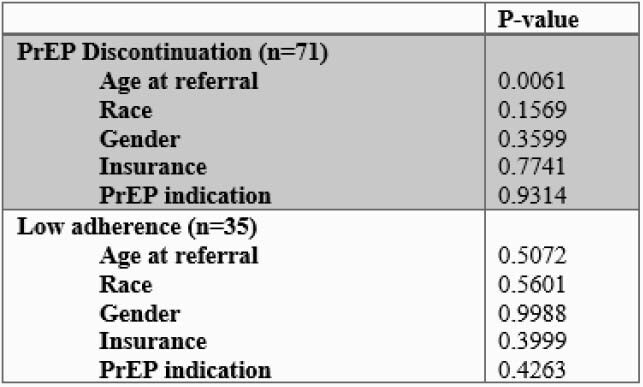

**Conclusion:**

Mean PrEP adherence at a safety net PrEP program in Atlanta was high and PrEP discontinuation rates were comparable to other PrEP clinics nationwide. We found no association with individual factors previously linked to lower adherence, including Black race, younger age, and insurance status. Program-related factors that may have impacted these findings need to be investigated. Other future areas of research include strategies to optimize engagement in care in younger patients.

**Disclosures:**

**Bradley L. Smith, Pharm.D., AAHIVP**, **Gilead Sciences, Inc** (Advisor or Review Panel member)

